# Primary Health Care structure and vaccination coverage in Brazilian municipalities

**DOI:** 10.11606/s1518-8787.2025059006279

**Published:** 2025-05-12

**Authors:** Guilherme de Andrade Ruela, Alaneir de Fátima dos Santos, César Macieira, Sábado Nicolau Girardi, Daisy Maria Xavier de Abreu, Alice Werneck Massote, Jackson Freire Araújo, Antônio Thomaz Gonzaga da Matta Machado

**Affiliations:** I Universidade Federal de Minas Gerais. Faculdade de Medicina. Programa de Pós-Graduação em Saúde Pública. Belo Horizonte, MG, Brasil; II Universidade Federal de Minas Gerais. Faculdade de Medicina. Núcleo de Educação em Saúde Coletiva. Belo Horizonte, MG, Brasil; III Escola de Saúde Pública do Estado de Minas Gerais. Belo Horizonte, MG, Brasil

**Keywords:** Vaccination Coverage, Primary Health Care, Structure of Services, Health Services Research, Quality of Health Care

## Abstract

**OBJECTIVES:**

To investigate the relationship between vaccination coverage indicators and the structure of primary care for immunization in Brazilian municipalities.

**METHODS:**

This was a time series ecological study using data from the National Immunization Program Information System (SI-PNI) and the National Program for Improving Primary Care Access and Quality (PMAQ) over three evaluation cycles. A total of 13 variables were assessed, five of which related to the structure of basic health units (BHU) and eight to the availability of immunobiologicals. Analyses of comparisons, associations, and longitudinal models were carried out to assess the influence of these indicators on vaccination coverage levels.

**RESULTS:**

The variables and indicators related to the structure of BHUs, the availability of immunobiologicals in Brazilian municipalities and vaccination coverage showed significant variations over the cycles. BHU structures ranged from fair to good, with lower percentages in Cycle 1 and increases in Cycles 2 and 3 for most of the variables analyzed. The availability of immunobiologicals also improved over the cycles, despite a few exceptions. Indicators of adequate vaccination coverage increased from Cycle 1 to Cycle 2 but decreased in Cycle 3. Improvements in the structure of the BHU and the availability of immunobiologicals were associated with higher adequate vaccination coverage. Keeping the availability of immunobiologicals fixed at good, the chance of having adequate coverage is 86.28% higher for a good structure compared to a poor one.

**CONCLUSIONS:**

Changes in the structure of municipal BHUs and in the availability of immunobiologicals over the cycles evaluated were identified and were associated with higher vaccination coverage when they occurred simultaneously (good availability of immunobiologicals and regular or good structure in BHUs). This highlights the importance of the quality of primary care in achieving vaccination coverage targets in Brazilian municipalities.

## INTRODUCTION

Active immunization, carried out through the administration of vaccines, plays a crucial role in the prevention and control of vaccine-preventable diseases, contributing significantly to global public health^1,2^, both by eradicating and reducing the incidence of these diseases^1,2^. To ensure the effectiveness of active immunization, it is essential to achieve the vaccination coverage rates (VCR) proposed by the National Immunization Program (PNI), which indicate the proportion of the target population vaccinated against certain diseases^3^. In Brazil, the expansion of VCRs has led to the eradication of poliomyelitis and a reduction of over 80.0% in the incidences of rubella, diphtheria, tetanus, and pertussis^1,2,4^.

Although it is desirable to achieve the proposed VCRs and keep them high, it is important to note that these rates often vary^1,5^, depending on the period evaluated^1,10^, the location^1,5^, the type of immunobiological (vaccine)^1,3^and the conditions of primary health care (PHC)^8,9,11^. In Brazil, the national average of the VCR, which was over 95.0% in 2015, began a downward trend with values below the recommended targets, as in 2019, when it reached only 46.0%^1,3,10^. It is worth noting that sometimes, even though the national LCI target is not reached, some regions, federal units, and municipalities manage to achieve them, or the opposite can happen. For example, in 2021, the Federal District and Amapá exceeded 95.0% of the infant VCR for BCG, while the national average was considered inadequate^3,10^. Among the factors that can influence this variation are: the training of health professionals^1,5^, the logistics of vaccine distribution and storage^1,9^, adverse socioeconomic factors^12^, the spread of fake news^12^, the anti-vaccine movement^12,13^, and above all the structure of PHC^6,7,13,14^and the availability of immunobiologicals in PHC^8,9,15^.

PHC is essential as an entry point into the health system, promoting, preventing, diagnosing, and rehabilitating the health of the population^16^. Its structure not only facilitates the efficient delivery of vaccination services, but also has a significant influence on community awareness, education and involvement in relation to immunization^13,14^. The structure of a PHC includes financial, human, and material resources, such as equipment and ambience^17^. As structural problems in PHC that affect the immunization service, some studies have observed that around a quarter of basic health units (BHU) did not have an exclusive environment for vaccination^16^and that those that did had structural problems, such as the color and permeability of the wall^16,17^. In addition to this, the ideal conditions for preserving immunobiologicals, which in most BHUs were carried out using thermal boxes, were often not considered satisfactory^18,19^.

The lack of ideal storage conditions, along with production and logistics problems, is a factor that can lead to the unavailability of immunobiologicals in PHC^9^. The availability of these immunobiologicals is crucial to achieving the proposed VCRs^1,7,9^. Although in Brazil there has been a growing trend in the prevalence of supply and availability of immunobiologicals over the years^8,15^, there are inconsistencies, especially according to the region of the country^1,8,9^, as some locations have greater availability than others^9,20^.

Thus, an integrated approach that considers the accessibility, acceptability and quality of the services provided is essential to achieving and sustaining high VCRs^20^. Therefore, an analysis that considers the various PHC factors, such as the exclusive vaccination environment, storage conditions, availability of immunobiologicals, among other aspects, is of paramount importance^7,12,20^. It is important to understand the importance and influence of these factors on VCRs over time and according to the regions of Brazil, since challenges in this interface can compromise collective health^21,22^.

For all the above reasons, this study aims to investigate the relationship between vaccination coverage indicators and the structure of primary care for immunization in Brazilian municipalities.

## METHODS

This is a temporal ecological study based on information about vaccination coverage and the structure of PHC, more specifically the BHU (structure for immunization and availability of immunobiologicals).

Vaccination coverage information was obtained from the National Immunization Program Information System (SI-PNI) database via Tabnet-Datasus (http://tabnet.datasus.gov.br/cgi/dhdat.exe?bd_pni/cpnibr.def - “Imunizações - Cobertura - Brasil”, [s.d.]). For the structure of the BHU for immunization (BHU structure), we used the records of the National Program for Improving Access and Quality in Primary Care (PMAQ) (https://www.gov.br/saude/pt-br/composicao/saps/pmaq). The PMAQ data corresponds to external evaluations carried out on site, from evaluation cycles 1 (Cycle 1), 2 (Cycle 2) and 3 (Cycle 3), which represent the periods 2011 to 2012, 2013 to 2015 and 2016 to 2019, respectively. The evaluations were carried out by accredited and trained interviewers, covering the BHU in each municipality throughout the country. The PMAQ data was transformed at municipal level by the Brazilian Institute of Geography and Statistics (IBGE) code to use the average number of teams/BHUs in the municipalities. For this study, we included municipalities that had more than 80.0% of the units/teams in at least two PMAQ cycles, which corresponded to 3,977 municipalities in Cycle 1; 3,947 municipalities in Cycle 2; and 3,962 municipalities in Cycle 3.

To ensure a reliable analysis, this study considered the variables present in the three PMAQ cycles. Thus, the variables analyzed in relation to the structure of the BHU were those pertaining to the existence of services, equipment and supplies, evaluated as a yes or no answer for: i. Vaccination service; ii. Vaccination service; ii. Vaccination room; iii. Refrigerator exclusively for vaccines; iv. Thermal boxes for vaccines; v. Vaccination cards. In relation to the availability of immunobiologicals, classified as “never/sometimes available” or “always available”, the variables analyzed were related to the vaccines: i. BCG-ID, ii. BCG-ID, ii. Hepatitis B, iii. Meningococcal C, iv. Poliomyelitis, v. Pneumococcal 10, vi. Triple viral, vii. Tetravalent/Pentavalent, and viii. Oral human rotavirus vaccine. The data contained information on the yellow fever vaccine, which was excluded due to the lack of data on vaccination coverage and its availability in many municipal BHU, as it is not a vaccine recommended throughout the country.

The variables “BHU structure” and “availability of immunobiologicals” were organized, categorized, and classified into three levels: good, fair, and bad. For “BHU structure”, a value of 0 was assigned for “no” and 1 for “yes” for the existence of each of the structure items (“vaccine room”, “refrigerator exclusively for vaccines”, “thermal boxes for vaccines”, and “vaccination card”), except for “vaccination service”, which received a value of 0 for “no” and 2 for “yes”, as it was considered the most crucial item. The BHU were thus classified as follows: Bad - zero to three items present (corresponding to 0 to 50.0%); Fair - four and five items present (51.0% to 79.0%); and Good - six and seven items present (80.0% to 100%).

As for the availability of immunobiologicals, a value of zero was assigned for “never/sometimes available” and a value of 1 for “always available”. The ratings were as follows: Bad - zero, one, two, three and four immunobiologicals always available (corresponding to less than 50.0%); Fair - five and six immunobiologicals always available (corresponding to more than 50.0% and less than 80.0%); and Good - seven and eight immunobiologicals always available (corresponding to 80.0% or more).

Regarding vaccination coverage, this was obtained annually from the SI-PNI, but in order to standardize the analyses according to the cycles evaluated in the PMAQ, the average vaccination coverage was obtained for the years of each cycle evaluated. Thus, the vaccination coverage analyzed referred to the vaccines selected in cycles 1 to 3 of the PMAQ, as mentioned above. The Tetravalent vaccine was considered in Cycle 1, while the Pentavalent vaccine was considered from 2013 onwards (Cycles 2 and 3). In the case of the triple viral vaccine, only Dose 1 (D1) was considered. Vaccination coverage was classified into three levels, according to the vaccination target: very low (0 to < 50.0%); low (greater than or equal to 50.0% and less than the target); and adequate (greater than or equal to the target), with the target being set by the Ministry of Health’s PNI. In addition, an overall score was given for vaccination coverage, with zero corresponding to very low coverage, one to low coverage and two to adequate coverage. The sum of these scores was assessed considering the frequency of vaccination coverage in the municipalities, categorized as follows: very low - zero to eight points (0 to 50.0%); low - nine to 13 points (51.0% to 84.0%); and adequate - 14 to 16 points (above 85.0%).

After adjustments were made to the database, they were analyzed using the R software version 4.3.0 and the significance level adopted was 5%. Categorical variables were expressed as absolute and relative frequencies and numerical variables were described by measures of central tendency and dispersion. The chi-square and Fisher’s exact tests were used to assess the association between categorical variables^23,24^. The Anderson-Darling test^25^ was used to check whether the data followed a normal distribution and once the hypothesis of normality was rejected, we used the Kruskal-Wallis test^26^, appropriate for non-parametric data, to analyze the difference between the PMAQ cycles (Cycle 1 to 3) in the municipalities. We then applied Nemenyi’s Multiple Comparisons^26^to identify specific differences between the individual methods.

In order to assess the influence of structural indicators and the availability of immunobiologicals on vaccination coverage levels ( “adequate” or “not adequate” general vaccination coverage score, with “not adequate” corresponding to the “very low” and “low” categories) , the logistic GEE (Generalized Equations Estimating) method^2 7^was used, since this is a longitudinal study that accounts for the correlation between repeated measures.

For the post-hoc analysis, the Estimated Marginal Means (EMM) technique was used^28^and pairwise comparisons were made using specified contrasts, allowing for a better understanding of the interactions between the variables. This technique helps to identify specific differences between the groups, clarifying the effects of interactions between the structure of the BHU and the availability of immunobiologicals.

The primary data from the PMAQ was obtained through a free and informed consent form and was submitted to and approved by Research Ethics Committees. For this research, secondary data from the SI-PNI and PMAQ, in the public domain and without identifying participants, were used, and it was not submitted to the Research Ethics Committee, in accordance with National Health Council Resolution (CNS) 510 of April 7, 2016.

## RESULTS

Analysis of the variables related to the structure of BHUs and the availability of immunobiologicals in Brazilian municipalities over the three evaluation cycles showed significant variations between the cycles (p-values < 0.001) considering all the variables analyzed (Table 1). The variables related to the structure of the BHU ranged from 62.4% (refrigerator exclusively for vaccines - Cycle 1) to 89.82% (vaccination card - Cycle 3), indicating a structure classified as fair to good. When analyzing each cycle individually, on average, it was observed that Cycle 1 had the lowest percentages for all the variables, showing the presence of a regular structure; on the other hand, Cycles 2 and 3 had higher percentages, with some variables reaching values above 80.0%, thus indicating a regular to good structure (Table 1). As for the availability of immunobiologicals, there was a wider range in the percentages, varying from 30.8% (Polio - Cycle 2) to 78.6% (Hepatitis B - Cycle 3). In Cycle 1, the availability of immunobiologicals was considered, on average, regular for most vaccines, with a range of 42.2% to 60.4%; in Cycles 2 and 3, the availability of immunobiologicals increased, except for the Polio vaccine in Cycle 2 and BCG-IG in Cycle 3. However, the overall classification remained regular, ranging from 30.8% to 78.6% (Table 1).

**Table 1:** Characterization of the variables related to the presence of structure in basic health units for immunization and the availability of immunobiologicals in basic health units in Brazilian municipalities in the three PMAQ evaluation cycles. Brazil, 2011 to 2019.

Variable evaluated	Cycle^a^ 1		Cycle 2		Cycle 3		p-value^b^
	n	%	n	%	n	%	
BHU structure for immunization (% of structure present)							
Vaccination service	2,898	72.87	3,236	81.99	3,267	82.54	< 0.001
Vaccination Room	2,537	63.79	3,093	78.36	2,830	71.50	< 0.001
Refrigerator exclusively for vaccines	2,478	62.40	2,913	73.80	2,735	69.10	< 0.001
Thermal boxes for vaccines	2,627	66.05	3,132	79.35	3,374	85.25	< 0.001
Vaccination card	3,001	75.46	3,409	86.37	3,555	89.82	< 0.001
Availability of immunobiologicals at BHU (% of vaccines always available)							
BCG-ID	2,296	42.27	1,783	54.83	2,015	49.09	< 0.001
Hepatitis B	2,403	60.42	2,953	74.82	3,114	78.68	< 0.001
Meningococcal C	2,341	58.86	2,933	74.31	3,023	76.38	< 0.001
Poliomyelitis	2,383	59.92	1,218	30.86	2,926	73.93	< 0.001
Pneumococcal 10	2,282	57.38	2,848	72.16	3,085	77.94	< 0.001
MMR - D1	2,202	55.37	2,906	73.63	3,058	77.26	< 0.001
Tetravalent/Pentavalent	2,182	54.87	2,947	74.66	2,994	75.64	< 0.001
Oral human rotavirus vaccine	2,378	59.79	2,940	74.49	2,807	70.92	< 0.001

PMAQ: Programa Nacional de Melhoria do Acesso e da Qualidade da Atenção Básica; n: corresponding number of municipalities; BHU: Basic Health Unit; BCC: bacillus Calmette and Guérin.

^a^ Cycle 1 corresponds to the period from 2011 to 2012, Cycle 2 from 2013 to 2015, and Cycle 3 from 2016 to 2019; ^b^ Chi-square test.

**Table 2:** Indicators of the structure of basic health units for immunization, availability of immunobiologicals and vaccination coverage scores in Brazilian municipalities according to PMAQ cycles 1, 2 and 3. Brazil, 2011 to 2019.

Variable evaluated	Indicator	Cycle^a^ 1		Cycle 2		Cycle 3		p-value^b^
		n	%	n	%	n	%	
Structure^c^	Bad	1,343	33.77	826	20.93	705	17.79	< 0.001
	Fair	618	15.54	474	12.01	1,025	25.87	
	Good	2,016	50.69	2,647	67.06	2,232	56.34	
Availability of immunobiologicals	Bad	1,602	40.28	996	25.23	885	22.34	< 0.001
	Fair	272	6.84	685	17.35	347	8.76	
	Good	2,103	52.88	2,266	57.41	2,730	68.90	
Overall vaccination coverage score	Very low	585	14.71	347	8.79	770	19.43	< 0.001
	Low	2,099	52.78	1,424	36.08	1,525	38.49	
	Adequate	1,293	32.51	2,176	55.13	1,667	42.07	
BCG-ID	Very low	727	18.28	711	18.01	420	10.60	< 0.001
	Low	1,280	32.19	983	24.90	1,421	35.87	
	Adequate	1,970	49.53	2,253	57.08	2,121	53.53	
Oral human rotavirus vaccine	Very low	36	0.91	25	0.63	29	0.73	< 0.001
	Low	1,615	40.61	843	21.36	1,161	29.30	
	Adequate	2,326	58.49	3,079	78.01	2,772	69.96	
Meningococcal C	Very low	44	1.11	15	0.38	28	0.71	< 0.001
	Low	1,098	27.61	966	24.47	1,541	38.89	
	Adequate	2,835	71.28	2,966	75.15	2,393	60.40	
Hepatitis B	Very low	4	0.10	18	0.46	45	1.14	< 0.001
	Low	1,401	35.23	990	25.08	1,750	44.17	
	Adequate	2,572	64.67	2,939	74.46	2,167	54.69	
Tetravalent/ Pentavalent	Very low	26	0.65	18	0.46	48	1.21	< 0.001
	Low	2,541	63.89	1,100	27.87	2,116	53.41	
	Adequate	1,410	35.45	2,829	71.67	1,798	45.38	
Pneumococcal 10	Very low	100	2.51	23	0.58	20	0.50	< 0.001
	Low	1,848	46.47	1,166	29.54	1,268	32.00	
	Adequate	2,029	51.02	2,758	69.88	2,674	67.49	
Poliomyelitis	Very low	9	0.23	21	0.53	28	0.71	< 0.001
	Low	1,350	33.95	1,064	26.96	1,891	47.73	
	Adequate	2,618	65.83	2,862	72.51	2,043	51.56	
MMR - D1	Very low	9	0.23	6	0.15	24	0.61	< 0.001
	Low	1,369	34.42	682	17.28	1,503	37.94	
	Adequate	2,599	65.35	3,259	82.57	2,435	61.46	

PMAQ: Programa Nacional de Melhoria do Acesso e da Qualidade da Atenção Básica; n: corresponding number of municipalities; BCC: bacillus Calmette and Guérin.

^a^ Cycle 1 corresponds to the period from 2011 to 2012, Cycle 2 from 2013 to 2015, and Cycle 3 from 2016 to 2019; ^b^ Chi-square test; ^c^ Structure of basic health units for immunization.

When evaluating the indicators of structure and availability of immunobiologicals, they varied over the cycles, but both indicators were considered to be good on average in all cycles. The availability of immunobiologicals increased over the cycles, being good in 52.8% of the municipalities in Cycle 1 and 68.9% in Cycle 3, with a significant increase in these indices over the cycles evaluated (p-values < 0.05). Good structure increased from Cycle 1 to Cycle 2, decreasing in Cycle 3. Regular structure increased in Cycle 3 (p-values < 0.05) (Table 2).

Like the structure and availability indicators, the indicators for the frequency of vaccination coverage, considered adequate, varied over the cycles (p-values < 0.0001). In Cycle 1, the lowest percentages of municipalities with adequate vaccination coverage were recorded, ranging from 35.4% (penta) to 71.2% (meningo C) (Table 2). In Cycle 2, the frequencies of municipalities with adequate vaccination coverage (in general and by immunobiological) increased, but there was a drop in these indicators in Cycle 3 (Table 2).

Individually analyzing the variables related to the presence of BHU structures, such as vaccination service, vaccination room, refrigerator exclusively for vaccines, thermal boxes for vaccines, and vaccination card, there were significant variations according to the vaccination coverage score indicator (p-values < 0.05), except for the vaccination service in Cycle 1 and the vaccination card in Cycle 3 (Table 3). The same fact was observed when considering the individual availability of immunobiologicals at the BHU, apart from the oral human rotavirus vaccine (Table 3). It should be noted that in most cycles, the BHUs that always had the immunobiologicals available had higher adequate vaccination coverage indicators (p-values < 0.05), except for BCG-ID in Cycle 1 and Polio in Cycle 2. Finally, in Cycle 3, all the parameters evaluated represented most municipalities for the adequate coverage score indicator.

**Table 3:** Evaluation of the structure of basic health units for immunization and the availability of immunobiologicals in relation to the general vaccination coverage score indicator in Brazilian municipalities, according to the PMAQ evaluation cycles. Brazil, 2011 to 2019.

Variable evaluated		Overall vaccination coverage score indicator														
		Cycle^a^ 1				p-value	Cycle 2				p-value	Cycle 3				p-value^b^
		Low		Adequate			Low		Adequate			Low		Adequate		
		n	%	n	%		n	%	n	%		n	%	n	%	
Structure^c^	Good	1,050	50.02	708	54.76	0.001	952	66.85	1,519	69.81	< 0.001	877	57.51	870	52.19	< 0.001
Availability of immunobiologicals		1,085	51.69	734	56.77	0.006	783	54.99	1,326	60.94	< 0.001	1,037	68.00	1,152	69.11	< 0.001
Vaccination service	Yes	1,534	73.08	935	72.31	0.854	1,201	84.34	1,754	80.61	0.015	1,257	82.53	1,331	79.84	< 0.001
Vaccination room		1,326	63.17	871	67.36	< 0.001	1,131	79.42	1,713	78.72	0.007	1,097	72.03	1,155	69.29	0.008
Refrigerator exclusively for vaccines		1,290	61.52	855	66.28	< 0.001	1,072	75.28	1,645	75.60	< 0.001	1,064	69.86	1,073	64.37	< 0.001
Thermal boxes for vaccines		1,373	65.41	910	70.38	< 0.001	1,138	79.92	1,756	80.70	< 0.001	1,313	86.21	1,388	83.26	0.007
Vaccination card		1,579	75.23	1010	78.11	0.002	1,245	87.43	1,870	85.94	0.286	1,381	90.68	1,460	87.58	< 0.001
BCG-ID	Always available	1,265	39.73	683	47.18	< 0.001	677	52.46	917	57.86	< 0.001	860	43.53	759	54.47	< 0.001
Hepatitis B		1,257	59.89	824	63.73	0.001	1,077	75.63	1,644	75.55	0.002	1,190	78.14	1,273	76.36	< 0.001
Meningococcal C		1,226	58.41	791	61.18	0.051	1,071	75.21	1,634	75.09	0.001	1,162	76.30	1,236	74.15	< 0.001
Poliomyelitis		1,244	59.27	815	63.03	0.005	411	28.86	731	33.59	< 0.001	1,121	73.60	1,207	72.41	0.016
Pneumococcal 10		1,197	57.03	778	60.17	0.007	1,044	73.31	1,588	72.98	< 0.001	1,176	77.22	1,264	75.82	< 0.001
Triple viral		1,144	54.50	762	58.93	0.002	1,057	74.23	1,622	74.54	0.001	1,175	77.15	1,247	74.81	< 0.001
Tetravalent Pentavalent		1,123	53.50	754	58.31	0.008	1,078	75.70	1,635	75.14	0.005	1,152	75.64	1,232	73.91	0.013
Oral human rotavirus vaccine		1,241	59.12	810	62.65	0.015	1,073	75.35	1,641	75.41	< 0.001	1,067	70.06	1,176	70.55	0.221

PMAQ: Programa Nacional de Melhoria do Acesso e da Qualidade da Atenção Básica; n: corresponding number of municipalities; BCC: bacillus Calmette and Guérin.

^a^ Cycle 1 corresponds to the period from 2011 to 2012, Cycle 2 from 2013 to 2015, and Cycle 3 from 2016 to 2019; ^b^ Kruskal-Wallis test; ^c^ Structure of basic health units for immunization.

There was an association between vaccination coverage scores (classified as very low, low and adequate) in the municipalities, federal units and regions of occurrence (p-values < 0.001). The behavior of the adequate level was the same in all regions, i.e. an increase from Cycle 1 to Cycle 2 and a decrease from Cycle 2 to Cycle 3 (Table 4). The regions with the highest vaccination coverage score indicators in municipalities classified as adequate were: South, Southeast, and Center-West (Table 4).

**Table 4:** Evaluation of the vaccination coverage indicator score in the municipalities (very low, low and adequate) according to the regions and states of Brazil throughout the PMAQ evaluation cycles. Brazil, 2011 to 2019.

Variable		Overall vaccination coverage score indicator																				
		Cycle^a^ 1						p-value	Cycle 2						p-value	Cycle 3						p-value^b^
		Very low		Low		Adequate			Very low		Low		Adequate			Very low		Low		Adequate		
		n	%	n	%	n	%		n	%	n	%	n	%		n	%	n	%	n	%	
Region	Center-West	35	9.38	177	47.45	161	43.16	< 0.001	12	3.23	106	28.49	254	68.28	< 0.001	76	20.32	134	35.83	164	43.85	< 0.001
	Northeast	297	23.80	711	56.97	240	19.23		225	18.01	568	45.48	456	36.51		370	29.58	494	39.49	387	30.94	
	North	52	20.63	136	53.97	64	25.40		32	12.85	108	43.37	109	43.78		64	24.90	95	36.96	98	38.13	
	Southeast	116	9.53	631	51.85	470	38.62		39	3.24	399	33.11	767	63.65		168	13.90	460	38.05	581	48.06	
	South	85	9.58	444	50.06	358	40.36		39	4.47	243	27.87	590	67.66		92	10.56	342	39.27	437	50.17	
State	AC	2	33.33	4	66.67	0	0.00	< 0.001	3	50.00	3	50.00	0	0.00	< 0.001	4	66.67	2	33.33	0	0.00	< 0.001
	AL	19	23.46	52	64.20	10	12.35		10	12.35	38	46.91	33	40.74		7	8.64	38	46.91	36	44.44	
	AM	12	52.17	11	47.83	0	0.00		3	13.04	15	65.22	5	21.74		12	52.17	8	34.78	3	13.04	
	AP	2	20.00	5	50.00	3	30.00		5	50.00	2	20.00	3	30.00		5	50.00	4	40.00	1	10.00	
	BA	76	25.25	170	56.48	55	18.27		55	18.33	133	44.33	112	37.33		145	48.33	90	30.00	65	21.67	
	EC	6	4.48	66	49.25	62	46.27		0	0.00	14	10.45	120	89.55		4	2.99	39	29.10	91	67.91	
	ES	1	2.13	28	59.57	18	38.30		0	0.00	8	17.02	39	82.98		4	8.51	19	40.43	24	51.06	
	GO	17	8.10	98	46.67	95	45.24		8	3.81	73	34.76	129	61.43		38	18.10	88	41.90	84	40.00	
	MA	9	28.13	21	65.63	2	6.25		4	12.50	13	40.63	15	46.88		10	31.25	13	40.63	9	28.13	
	MG	81	11.71	341	49.28	270	39.02		24	3.50	211	30.80	450	65.69		80	11.58	243	35.17	368	53.26	
	MS	2	3.57	31	55.36	23	41.07		1	1.79	4	7.14	51	91.07		15	26.32	14	24.56	28	49.12	
	MT	16	14.95	48	44.86	43	40.19		3	2.83	29	27.36	74	69.81		23	21.50	32	29.91	52	48.60	
	PA	14	20.00	44	62.86	12	17.14		9	13.24	42	61.76	17	25.00		35	52.24	22	32.84	10	14.93	
	PB	84	42.42	105	53.03	9	4.55		48	24.24	107	54.04	43	21.72		43	21.72	113	57.07	42	21.21	
	PE	24	16.00	90	60.00	36	24.00		12	8.00	81	54.00	57	38.00		19	12.67	78	52.00	53	35.33	
	PI	44	28.21	99	63.46	13	8.33		71	44.10	78	48.45	12	7.45		68	42.24	54	33.54	39	24.22	
	PR	16	5.50	137	47.08	138	47.42		2	0.69	71	24.48	217	74.83		24	8.33	102	35.42	162	56.25	
	RJ	2	3.17	45	71.43	16	25.40		1	1.61	20	32.26	41	66.13		7	11.11	37	58.73	19	30.16	
	RN	33	23.74	83	59.71	23	16.55		22	16.06	86	62.77	29	21.17		63	45.32	47	33.81	29	20.86	
	RO	1	3.45	19	65.52	9	31.03		1	3.23	11	35.48	19	61.29		0	0.00	10	31.25	22	68.75	
	RR	4	66.67	2	33.33	0	0.00		0	0.00	5	83.33	1	16.67		1	16.67	2	33.33	3	50.00	
	RS	57	16.96	169	50.30	110	32.74		24	7.45	84	26.09	214	66.46		47	14.46	126	38.77	152	46.77	
	SC	12	4.62	138	53.08	110	42.31		13	5.00	88	33.85	159	61.15		21	8.14	114	44.19	123	47.67	
	SE	2	3.51	25	43.86	30	52.63		3	5.36	18	32.14	35	62.50		11	19.64	22	39.29	23	41.07	
	SP	32	7.71	217	52.29	166	40.00		14	3.41	160	38.93	237	57.66		77	18.87	161	39.46	170	41.67	
	TO	17	15.74	51	47.22	40	37.04		11	10.48	30	28.57	64	60.95		7	6.19	47	41.59	59	52.21	

PMAQ: Programa Nacional de Melhoria do Acesso e da Qualidade da Atenção Básica; n: corresponding number of municipalities; AC (Acre); AL (Alagoas); AM (Amazonas); AP (Amapá); BA (Bahia); CE (Ceará); ES (Espírito Santo); GO (Goiás); MA (Maranhão); MG (Minas Gerais); MS (Mato Grosso do Sul); MT (Mato Grosso); PA (Pará); PB (Paraíba); PE (Pernambuco); PI (Piauí); PR (Paraná); RJ (Rio de Janeiro); RN (Rio Grande do Norte); RO (Rondônia); RR (Roraima); RS (Rio Grande do Sul); SC (Santa Catarina); SE (Sergipe); SP (São Paulo); TO (Tocantins).

^a^ Cycle 1 corresponds to the period from 2011 to 2012, Cycle 2, from 2013 to 2015, and Cycle 3, from 2016 to 2019.

^b^ Chi-square and Fisher’s exact tests.

In Cycle 1, only Sergipe had more than 50.0% of its municipalities with this indicator. In Cycle 2, 14 states had most of their municipalities with an adequate coverage score, with Mato Grosso do Sul standing out (91.07%). Finally, in Cycle 3, only seven states had 50.0% or more of their municipalities with an adequate indicator, with the highest frequency being seen in Rondônia (68.75%) (Table 4). Looking at the municipal level, it is worth noting that throughout the PMAQ cycles evaluated, no municipality in Acre had an adequate vaccination coverage indicator score, nor did the municipalities in Amazonas and Roraima (Cycle 1).

When investigating the relationship between the indicators of structure for immunization and availability of immunobiologicals and their effects on vaccination coverage (adequate or not adequate), it was observed that improving the structure of the BHU or the availability of immunobiologicals results in greater vaccination coverage (Table 5). Keeping the “availability of immunobiologicals” indicator fixed (good), the chance of having adequate coverage is 86.28% (= 3.22%+ 83.06%) higher than inadequate coverage for a good structure indicator compared to a poor one (p-value< 0.05). As for good availability in relation to poor availability, keeping the “structure” indicator fixed (good), the chance of having adequate coverage is 48.3% (= -34.76%+ 83.06%), higher than the non-adequate coverage for a good “immunobiological availability” indicator in relation to a poor “immunobiological availability” indicator (p-value< 0.05) (Table 5).

**Table 5:** Analysis of the influence of basic health units and immunobiologicals on vaccination coverage. Brazil, 2011 to 2019.

Generalized estimating equations							
Variable	β	Error Standard	Exp(β)	Amendment	95%CI Exp(β)	95%CI Change	p-value
EST = Bad	-	-	1,00	-	-	-	-
EST = Fair	-0.25	0.10	0.78	-22.07%	(0.639 to 0.95)	(-36.1% to -4.95%)	0.014
EST = Good	0.03	0.13	1.03	3.22%	(0.793 to 1.343)	(-20.66% to 34.3%)	0.813
DI = Bad	-	-	1.00	-	-	-	-
DI = Fair	-0.19	0.36	0.83	-16.94%	(0.412 to 1.675)	(-58.81% to 67.51%)	0.604
DI = Good	-0.43	0.27	0.65	-34.76%	(0.385 to 1.106)	(-61.51% to 10.58%)	0.113
EST = Fair* DI = Fair	0.44	0.38	1.55	55.05%	(0.731 to 3.291)	(-26.94% to 229.06%)	0.253
EST = Good* DI = Fair	0.21	0.38	1.24	23.55%	(0.583 to 2.618)	(-41.69% to 161.77%)	0.581
EST = Fair* DI = Good	0.71	0.29	2.03	102.76%	(1.147 to 3.584)	(14.71% to 258.39%)	0.015
EST = Good* DI = Good	0.60	0.30	1.83	83.06%	(1.016 to 3.297)	(1.64% to 229.69%)	0.044
**Marginal estimates of averages**							
**Indicator availability of immunobiologicals**	**Indicator structure of the BHU**		**Estimate**		**Error**	**p-value**	
Bad - Fair	Structure = Bad		0.186		0.358	0.604	
Bad - Good	Structure = Bad		0.427		0.269	0.113	
Fair - Good	Structure = Bad		0.242		0.448	0.590	
Bad - Fair	Structure = Regular		-0.253		0.143	0.077	
Bad - Good	Structure = Regular		-0.280		0.108	0.009	
Fair - Good	Structure = Regular		-0.027		0.122	0.826	
Bad - Fair	Structure = Good		-0.026		0.144	0.857	
Bad - Good	Structure = Good		-0.178		0.131	0.175	
Fair - Good	Structure = Good		-0.152		0.068	0.026	

BHU: basic health unit; 95%CI: 95% confidence interval; EST: structure of basic health units for immunization; DI: availability of immunobiologicals.

The contrasts show that good availability of immunobiologicals significantly favors vaccination coverage compared to poor availability (p-value< 0.01). For BHUs with a good structure, the availability of both regular and good immunobiologicals increases vaccination coverage (Table 5).

## DISCUSSION

The National Primary Health Care Policy (PNAB) emphasizes the importance of infrastructure compliance in BHUs (the existence of a vaccination room is recommended), which must comply with sanitary standards, as well as having the appropriate ambience, adequate equipment, trained human resources and sufficient materials and supplies for health care^29^. This is essential to guarantee adequate conditions for the full operation of these units. In this study, the analysis of structure indicators showed that, in general, there were improvements in the structures of municipal BHUs between the PMAQ cycles evaluated (Cycle 1 - from 2011 to 2012; Cycle 2 - from 2013 to 2015; and Cycle 3 - from 2016 to 2019). Variations in BHU structures have already been observed^15,17^, including when considering evaluation cycles^7,17^, and can be explained by different factors, such as investments in infrastructure^17^, changes in guidelines^17,18^and health policies^22,29^. The same factors are related to the improvements observed in the availability of immunobiologicals in municipal BHUs over the PMAQ cycles evaluated^8^.

The availability of immunobiologicals is linked to the timeliness of vaccination and is influenced by various factors such as: ideal storage conditions, production and distribution logistics^8,9,16^. It is estimated that one third of children in low- and middle-income countries have not received the vaccines on the basic calendar for the first year of life, which is a missed vaccination opportunity. It is therefore important to look separately at the availability of each immunobiological evaluated in this study, as well as its storage conditions (refrigerators and/or thermal boxes). When we looked at this item for each immunobiological, we found high availability in Cycles 2 and 3, with values of over 70.0%, except for the BCG-ID vaccine, which showed low availability in all the cycles evaluated (< 55.0%), and similar results have been observed in other studies^8,19^.

Despite the significant increase in the availability of vaccines over the cycles in this study, it is important to note that there has been a drop in the availability of most of the vaccines on the childhood vaccination schedule^8^, a fact that compromises the health of both children and the general population^8,30^. It should be noted that “the irregularity in the supply of immunobiologicals due to production problems, both related to the production process of public and private laboratories, identified in recent years”^1^can influence the monitoring of the VCI, either due to the lack of timely vaccination or, even if the shortage is regularized, depending on the age group, the dose administered at a later time (outside the specific age group) will not count towards the indicator calculations^1^. The inclusion of new vaccines over time in the country’s vaccination schedule can also influence the availability of these immunobiologicals and their coverage, considering the time it takes for vaccination services throughout the country to have access to these products, since since 2006, “there has been a growing incorporation of new vaccines into the PNI”^1^.

Regarding VCRs, there were municipalities with higher VCRs in Cycle 2, followed by a significant drop, resulting in levels below the vaccination targets recommended by the Ministry of Health, which can be seen from 2016^1^. The reduction in the VCR is cause for concern as it is associated with an increase in the incidence of vaccine-preventable diseases. An example of this is the return of measles, a disease from which Brazil had been declared free since 2016, but which in 2018 recorded more than 10,000 cases^1^. It is believed that this drop in vaccination during Cycle 3 is mainly related to adverse socio-economic factors^14^, the spread of fake news^15^, the anti-vaccine movement^12,16^and the decreased emphasis on prevention, due to the reduction in the incidence of various diseases^1^, as well as the structure of the BHU and their supply of immunobiologicals^1,8,9^.

The adequacy of the BHU structure was associated with higher VCRs, a fact that corroborates the results of other studies^7,14,15,30^, as well as the availability of immunobiologicals^8,9,15^. Curiously, when the structures of municipal BHUs are considered regular, there is a reduction in vaccination coverage. These results diverge from expectations and indicate the need for more in-depth research into the determinants of vaccination coverage and its relationship with the structure and availability of immunobiologicals in BHUs, since even if the situation is regular, it should be better than a poor structure. However, when evaluating the association of these factors together in terms of the LCI, it can be seen that when the availability of immunobiologicals is classified as good and the structure of the BHU as regular or good, there is a significant increase in vaccination coverage. This suggests that the combination of these factors can have a positive impact on promoting vaccination and improving the health of the population, highlighting the importance of analyzing them together in order to better understand the determinants of vaccination coverage.

When considering the VCR according to the regions of Brazil, it can be seen that the increases in the VCR over the cycles evaluated in the North, Center-West and Northeast regions had already been observed in the literature^8^. These increases were associated with greater vaccine availability and government initiatives to reduce inequality between health services^8^. Historically, these regions have had the lowest vaccination coverage, and the causes are often associated with social and development indicators^31^. On the other hand, it is believed that the reduction in VCRs over the same period in the South and Southeast is mainly due to the spread of fake news^12^and the anti-vaccine movement^12,16^. These factors contribute to a decrease in confidence in vaccination and can have a negative impact on efforts to maintain high vaccination coverage rates in these regions.

It is important to highlight some of the limitations of this study. With regard to the vaccination coverage provided by the SI-PNI, there may be problems with the records, with coverage being underestimated or overestimated. Municipal SI-PNI coverage may include services outside the BHU. The PMAQ analysis was restricted to the immunization structures located within the BHU that joined the program and that had more than 80.0% of the units/teams participating in at least two PMAQ cycles, which, in most cases, are considered to be of better quality^19,32^. In addition, information on centralized immunization structures in the municipalities was not considered. Therefore, it is necessary to be cautious when generalizing the results obtained for all PHC services in Brazil, given that in 2019 alone there were more than 43,000 family health teams (ESF) distributed in 5,476 Brazilian municipalities (around 0.98 ESF/municipality)^33^.

In view of the above, it can be concluded that there have been improvements in the structure of the municipal BHUs evaluated and an increase in the availability of immunobiologicals over time. Vaccination coverage indicators in the municipalities varied over the years and according to the regions/states of Brazil. It was noted that higher indicators were observed when there was a combination of good availability of immunobiologicals and regular or good structure in the BHUs, highlighting the importance of more comprehensive investigations into the determinants of vaccination in different territories, quality improvement and the role of primary care in promoting not only immunization, but also the reduction of health inequalities.
