# Supplementary material for: Primary Health Care structure and vaccination coverage in Brazilian municipalities
Source: Rev Saude Publica. 2025 May 12;59:e12. doi: 10.11606/s1518-8787.2025059006279 (PMC12077761; doi:10.11606/s1518-8787.2025059006279)
Supplement: Translation

# Translation: Estrutura da Atenção Primária à Saúde e as coberturas vacinais nos municípios brasileiros

**Authors:** Guilherme de Andrade Ruela, Alaneir de Fátima dos Santos, César Macieira, Sábado Nicolau Girardi, Daisy Maria Xavier de Abreu, Alice Werneck Massote, Jackson Freire Araújo, Antônio Thomaz Gonzaga da Matta Machado

**Affiliations:** I Universidade Federal de Minas Gerais. Faculdade de Medicina. Programa de Pós-Graduação em Saúde Pública. Belo Horizonte, MG, Brasil; II Universidade Federal de Minas Gerais. Faculdade de Medicina. Núcleo de Educação em Saúde Coletiva. Belo Horizonte, MG, Brasil; III Escola de Saúde Pública do Estado de Minas Gerais. Belo Horizonte, MG, Brasil

**Keywords:** Cobertura Vacinal, Atenção Primária à Saúde, Estrutura dos Serviços, Avaliação de Serviços de Saúde, Qualidade dos Serviços de Saúde

## Abstract

**OBJETIVO:**

Investigar a relação entre os indicadores de cobertura vacinal e a estrutura da atenção primária para imunização nos municípios brasileiros.

**MÉTODOS:**

Trata-se de um estudo ecológico temporal que utilizou dados do Sistema de Informação do Programa Nacional de Imunizações (SI-PNI) e do Programa Nacional de Melhoria do Acesso e da Qualidade da Atenção Básica (PMAQ) ao longo de três ciclos de avaliação. Ao todo, foram avaliadas 13 variáveis, sendo cinco relacionadas à estrutura das unidades básicas de saúde (UBS) e oito à disponibilidade de imunobiológicos. Foram realizadas análises de comparações, associações e modelos longitudinais para avaliar a influência desses indicadores nos níveis de cobertura vacinal.

**RESULTADOS:**

As variáveis e os indicadores relacionados à estrutura das UBS, à disponibilidade de imunobiológicos nos municípios brasileiros e às coberturas vacinais apresentaram variações significativas ao longo dos ciclos. As estruturas das UBS variaram de regular a boa, com percentuais mais baixos no Ciclo 1 e aumentos nos Ciclos 2 e 3 para a maioria das variáveis analisadas. A disponibilidade de imunobiológicos também melhorou ao longo dos ciclos, apesar de algumas exceções. Os indicadores de cobertura vacinal adequada aumentaram do Ciclo 1 para o Ciclo 2, mas diminuíram no Ciclo 3. Melhorias na estrutura das UBS e na disponibilidade de imunobiológicos foram associadas a maiores coberturas vacinais adequadas. Mantendo a disponibilidade de imunobiológicos fixa como boa, a chance de ter cobertura adequada é 86,28% maior para uma estrutura boa em comparação a uma ruim.

**CONCLUSÕES:**

Mudanças na estrutura das UBS municipais e na disponibilidade de imunobiológicos ao longo dos ciclos avaliados foram identificas e elas foram associadas a uma maior cobertura vacinal quando ocorreram simultaneamente (boa disponibilidade de imunobiológicos e estrutura regular ou boa nas UBS). Destaca-se a importância da qualidade da atenção primária para o alcance das metas de cobertura vacinal nos municípios brasileiros.

## INTRODUÇÃO

A imunização ativa, realizada por meio da administração de vacinas, desempenha um papel crucial na prevenção e no controle de doenças imunopreveníveis, contribuindo significativamente para a saúde pública global^1,2^, tanto por meio da erradicação quanto pela redução na incidência dessas doenças^1,2^. Para assegurar a eficácia da imunização ativa, é fundamental alcançar os índices de cobertura vacinal (ICV) propostos pelo Programa Nacional de Imunizações (PNI), os quais indicam a proporção da população-alvo vacinada contra determinadas patologias^3^. No Brasil, a expansão dos ICV promoveu a erradicação da poliomielite e redução superior a 80,0% nas incidências de rubéola, difteria, tétano e coqueluche^1,2,4^.

Embora seja desejável alcançar os ICV propostos e mantê-los elevados, é importante observar que esses índices frequentemente variam^1,5^, dependendo do período avaliado^1,10^, da localidade^1,5^, do tipo de imunobiológico (vacina)^1,3^ e das condições da atenção primária à saúde (APS)^8,9,11^. No cenário brasileiro, a média nacional do ICV, que foi superior a 95,0% em 2015, iniciou uma tendência de queda com valores abaixo das metas preconizadas, como em 2019, quando alcançou apenas 46,0%^1,3,10^. Vale destacar que, às vezes, apesar de a meta nacional de ICV não ser alcançada, algumas regiões, unidades federativas e municípios conseguem atingi-las, ou o inverso pode acontecer. Por exemplo, em 2021, o Distrito Federal e o Amapá superaram 95,0% de ICV infantil para a BCG, enquanto a média nacional foi considerada inadequada^3,10^. Dentre os fatores que podem influenciar essa variação, destacam-se: a capacitação dos profissionais de saúde^1,5^, a logística de distribuição e armazenamento das vacinas^1,9^, os fatores socioeconômicos adversos^12^, a disseminação de notícias falsas^12^, o movimento antivacina^12,13^e, principalmente, a estrutura da APS^6,7,13,14^ e a disponibilidade de imunobiológico na APS^8,9,15^.

A APS é essencial como ponto de entrada no sistema de saúde, promovendo, prevenindo, diagnosticando e reabilitando a saúde da população^16^. Sua estrutura não apenas facilita a entrega eficiente dos serviços de vacinação, mas também exerce influência significativa na conscientização, na educação e no envolvimento da comunidade em relação à imunização^13,14^. A estrutura de uma APS compreende recursos financeiros, humanos e materiais, como, neste último caso, equipamentos e ambiência^17^. Como problemas estruturais na APS que afetam o serviço de imunização, alguns estudos observaram que cerca de um quarto das unidades básicas de saúde (UBS) não apresentavam ambiente exclusivo para a vacinação^16^e que aquelas que possuíam enfrentavam problemas estruturais, como a cor e a permeabilidade da parede^16,17^. Somado a isto, as condições ideais de conservação dos imunobiológicos, realizadas na maioria das UBS por meio de caixas térmicas, não foram, em muitas ocasiões, consideradas satisfatórias^18,19^.

A falta de condições ideais de conservação, juntamente a problemas de produção e de logística, é fator que pode ocasionar a indisponibilidade de imunobiológicos na APS^9^. A disponibilidade desses imunobiológicos é crucial para alcançar os ICV propostos^1,7,9^. Embora no Brasil exista uma tendência crescente da prevalência de fornecimento e disponibilidade dos imunobiológicos ao longo dos anos^8,15^, observam-se inconsistências, especialmente segundo a região do país^1,8,9^, já que algumas localidades apresentam maior disponibilidade dos que outras^9,20^.

Dessa forma, uma abordagem integrada que considere a acessibilidade, aceitabilidade e qualidade dos serviços prestados é essencial para alcançar e sustentar altos ICV^20^. Logo, uma análise que considere os diversos fatores da APS, como ambiente exclusivo de vacinação, condições de armazenamento, disponibilidade de imunobiológicos, entre outros aspectos é de suma importância^7,12,20^. É importante compreender a importância e a influência desses fatores nos ICV ao longo do tempo e conforme as regiões do Brasil, uma vez que desafios nessa interface podem comprometer a saúde coletiva^21,22^.

Por todo o exposto, este estudo objetiva investigar a relação entre os indicadores de cobertura vacinal e a estrutura da atenção primária para imunização nos municípios brasileiros.

## MÉTODOS

Trata-se de um estudo ecológico temporal que se baseou em informações sobre cobertura vacinal e estrutura da APS, mais especificamente das UBS (estrutura para imunização e disponibilidade de imunobiológicos).

As informações de cobertura vacinal foram obtidas a partir da base de dados do Sistema de Informação do Programa Nacional de Imunizações (SI-PNI) pelo Tabnet-Datasus (http://tabnet.datasus.gov.br/cgi/dhdat.exe?bd_pni/cpnibr.def - “Imunizações - Cobertura - Brasil”, [s.d.]). Para a estrutura das UBS para imunização (estrutura das UBS), foram utilizados os registros do Programa Nacional de Melhoria do Acesso e da Qualidade da Atenção Básica (PMAQ) (https://www.gov.br/saude/pt-br/composicao/saps/pmaq). Os dados do PMAQ correspondem a avaliações externas realizadas *in loco*, dos ciclos de avaliação 1 (Ciclo 1), 2 (Ciclo 2) e 3 (Ciclo 3) que representam, respectivamente, aos períodos de 2011 a 2012, de 2013 a 2015 e de 2016 a 2019. As avaliações foram realizadas por entrevistadores credenciados e treinados, abrangendo as UBS em cada município em todo o território nacional. Os dados do PMAQ foram transformados a nível municipal por código do Instituto Brasileiro de Geografia e Estatística (IBGE) para uso da média das equipes/UBS dos municípios. Para esse estudo, foram incluídos os municípios que tiveram a participação acima de 80,0% das unidades/equipes em pelo menos dois ciclos do PMAQ, o que correspondeu a 3.977 municípios no Ciclo 1; 3.947 municípios no Ciclo 2 e 3.962 municípios no Ciclo 3.

Para garantir uma análise confiável, este estudo considerou as variáveis presentes nos três ciclos do PMAQ. Assim, as variáveis analisadas em relação à estrutura das UBS foram aquelas pertinentes à existência de serviços, equipamentos e insumos, avaliadas como resposta sim ou não para: i. Serviço de vacinação; ii. Sala de Vacinação; iii. Geladeira exclusiva para vacinas; iv. Caixas térmicas para vacinas; v. Cartão de vacinação. Em relação à disponibilidade de imunobiológicos, classificadas como “nunca/às vezes disponível” ou “sempre disponível”, as variáveis analisadas foram relacionadas as vacinas: i. BCG-ID, ii. Hepatite B, iii. Meningocócica C, iv. Poliomielite, v. Pneumocócica 10, vi. Tríplice viral, vii. Tetravalente/Pentavalente e, viii. Vacina oral de rotavírus humano. Os dados continham informações referentes à vacina de febre amarela, as quais foram excluídas devido à ausência de dados quanto à cobertura vacinal e a sua disponibilidade em muitas UBS municipais, por não ser uma vacina recomendada em todo o território nacional.

As variáveis “estrutura da UBS” e “disponibilidade de imunobiológicos” foram organizadas, categorizadas e classificadas em três níveis: bom, regular e ruim. Para a “estrutura da UBS”, atribuiu-se o valor 0 para “não” e o valor 1 para “sim” para a existência de cada um dos itens de estrutura (“sala de vacina”, “geladeira exclusiva para vacina”, “caixas térmicas para vacina” e “cartão de vacinação”), exceto para “serviço de vacinação”, que recebeu o valor 0 para “não” e 2 para “sim”, por ser considerado o item mais crucial. Dessa forma, as UBS foram classificadas da seguinte maneira: Ruim – zero a três itens presentes (correspondendo a 0 a 50,0%); Regular – quatro e cinco itens presentes (51,0% a 79,0%); e Bom – seis e sete itens presentes (80,0% a 100%).

Quanto à disponibilidade de imunobiológicos, foi atribuído o valor zero para “nunca/às vezes disponível” e o valor 1 para “sempre disponível”. As classificações foram as seguintes: Ruim – zero, um, dois, três e quatro imunobiológicos sempre disponíveis (correspondendo a menos de 50,0%); Regular – cinco e seis imunobiológicos sempre disponíveis (correspondendo a mais de 50,0% e menos de 80,0%); e Bom – sete e oito imunobiológicos sempre disponíveis (correspondendo a 80,0% ou mais).

No que diz respeito às coberturas vacinais, estas foram obtidas de forma anual no SI-PNI, mas, visando padronizar as análises segundo os ciclos avaliados no PMAQ, obteve-se as médias das coberturas vacinais referentes aos anos de cada ciclo avaliado. Dessa forma, as coberturas vacinais analisadas foram referentes às vacinas selecionadas nos ciclos 1 a 3 do PMAQ, conforme mencionado anteriormente. A vacina Tetravalente foi considerada no Ciclo 1, enquanto a Pentavalente foi considerada a partir de 2013 (Ciclos 2 e 3). No caso da vacina tríplice viral, apenas a Dose 1 (D1) foi considerada. As coberturas vacinais foram classificadas em três níveis, de acordo com a meta de vacinação: muito baixa (0 a < 50,0%); baixa (maior ou igual a 50,0% e menor que a meta); e adequada (maior ou igual à meta), sendo a meta estabelecida pelo PNI do Ministério da Saúde. Ademais, foi atribuída uma pontuação geral para a cobertura vacinal, com zero correspondendo à cobertura muito baixa, um à cobertura baixa e dois à cobertura adequada. A soma dessas pontuações foi avaliada considerando a frequência das coberturas vacinais nos municípios, categorizadas da seguinte forma: muito baixa – zero a oito pontos (0 a 50,0%); baixa – nove a 13 pontos (51,0% a 84,0%); e adequada – 14 a 16 pontos (acima de 85,0%).

Após os ajustes realizados na base de dados, eles foram analisados no *software* R versão 4.3.0 e o nível de significância adotado foi de 5%. As variáveis categóricas foram expressas em frequências absoluta e relativa e as variáveis numéricas foram descritas por medidas de tendência central e dispersão. Para avaliar a associação entre as variáveis categóricas, foram usados os testes qui-quadrado e exato de Fisher^23,24^. O teste de Anderson-Darling^25^ foi utilizado para verificar se os dados seguiam uma distribuição normal e uma vez que a hipótese de normalidade foi rejeitada, utilizamos o teste de Kruskal-Wallis^26^, apropriado para dados não paramétricos, para analisar a diferença entre os ciclos do PMAQ (Ciclo 1 ao 3) nos municípios. Posteriormente, aplicou-se as Comparações Múltiplas de Nemenyi^26^ para identificar diferenças específicas entre os métodos individuais.

Para avaliar a influência dos indicadores de estrutura e a disponibilidade de imunobiológicos nos níveis de cobertura vacinal (pontuação de cobertura vacina geral “adequada” ou “não adequada”, sendo a “não adequada” correspondendo às categorias “muito baixa” e “baixa”), foi utilizado o método GEE (*Generalized Equations Estimating*) logístico^27^, uma vez que se trata de um estudo longitudinal que contabiliza a correlação existente entre as medidas repetidas.

Para a análise pós-hoc, utilizou-se a técnica de estimativas marginais das médias (EMM, *Estimated Marginal Means*)^28^ e foram realizadas comparações par a par utilizando contrastes especificados, permitindo entender melhor as interações entre as variáveis. Esta técnica ajuda a identificar diferenças específicas entre os grupos, esclarecendo os efeitos das interações entre a estrutura das UBS e a disponibilidade de imunobiológicos.

Os dados primários do PMAQ foram obtidos por meio de termo de consentimento livre e esclarecido, sendo submetidos e aprovados em Comitês de Ética em Pesquisa. Para esta pesquisa, foram utilizados dados secundários do SI-PNI e PMAQ, de domínio público e sem identificação de participantes, não sendo submetida ao Comitê de Ética em Pesquisa, conforme a Resolução do Conselho Nacional de Saúde (CNS) 510, de 7 de abril de 2016.

## RESULTADOS

A análise das variáveis relacionadas à estrutura das UBS e à disponibilidade de imunobiológicos nos municípios brasileiros ao longo dos três ciclos de avaliação apresentou variações significativas entre os ciclos (valores-p < 0,001) considerando todas as variáveis analisadas (Tabela 1). As variáveis relacionadas à estrutura das UBS variaram de 62,4% (geladeira exclusiva para vacina – Ciclo 1) a 89,82% (cartão de vacinação – Ciclo 3), indicando estrutura classificada como regular a boa. Ao analisar individualmente cada ciclo, em média, observou-se que o Ciclo 1 apresentou os menores percentuais, para todas as variáveis, mostrando a presença de uma estrutura regular; por outro lado, os Ciclos 2 e 3 apresentaram percentuais mais elevados, com algumas variáveis atingindo valores superiores a 80,0%, indicando, assim, uma estrutura de regular a boa (Tabela 1). Já em relação à disponibilidade dos imunobiológicos, observou-se uma amplitude maior nos percentuais, variando de 30,8% (Poliomielite – Ciclo 2) a 78,6% (Hepatite B – Ciclo 3). No Ciclo 1, a disponibilidade dos imunobiológicos foi considerada, em média, regular para a maioria das vacinas, com variação de 42,2% a 60,4%; nos Ciclos 2 e 3, a disponibilidade dos imunobiológicos aumentou, exceto para a vacina Poliomielite no Ciclo 2 e BCG-IG no Ciclo 3. Entretanto, a classificação geral permaneceu como regular, variando de 30,8% a 78,6% (Tabela 1).

**Tabela 1:** Caracterização das variáveis relacionadas à presença de estrutura das unidades básicas de saúde para imunização e à disponibilidade de imunobiológicos nas unidades básicas de saúde nos municípios brasileiros nos três ciclos de avaliação do PMAQ. Brasil, 2011 a 2019.

Variável avaliada	Ciclo^a^ 1		Ciclo 2		Ciclo 3		Valor-p^b^
	n	%	n	%	n	%	
Estrutura das UBS para imunização (% de estrutura presente)							
Serviço de vacinação	2.898	72,87	3.236	81,99	3.267	82,54	< 0,001
Sala de Vacina	2.537	63,79	3.093	78,36	2.830	71,50	< 0,001
Geladeira exclusiva para vacina	2.478	62,40	2.913	73,80	2.735	69,10	< 0,001
Caixas térmicas para vacinas	2.627	66,05	3.132	79,35	3.374	85,25	< 0,001
Cartão de vacinação	3.001	75,46	3.409	86,37	3.555	89,82	< 0,001
Disponibilidade de imunobiológico nas UBS (% de vacinas sempre disponíveis)							
BCG-ID	2.296	42,27	1.783	54,83	2.015	49,09	< 0,001
Hepatite B	2.403	60,42	2.953	74,82	3.114	78,68	< 0,001
Meningocócica C	2.341	58,86	2.933	74,31	3.023	76,38	< 0,001
Poliomielite	2.383	59,92	1.218	30,86	2.926	73,93	< 0,001
Pneumocócica 10	2.282	57,38	2.848	72,16	3.085	77,94	< 0,001
Tríplice viral - D1	2.202	55,37	2.906	73,63	3.058	77,26	< 0,001
Tetravalente/Pentavalente	2.182	54,87	2.947	74,66	2.994	75,64	< 0,001
Vacina oral de rotavírus humano	2.378	59,79	2.940	74,49	2.807	70,92	< 0,001

PMAQ: Programa Nacional de Melhoria do Acesso e da Qualidade da Atenção Básica; n: número de municípios correspondente; UBS: unidade básica de saúde; BCG: bacilo de Calmette e Guérin.

^a^ Ciclo 1 corresponde ao período de 2011 a 2012, o Ciclo 2, de 2013 a 2015, e o Ciclo 3, de 2016 a 2019; ^b^ Teste Qui-quadrado.

Ao avaliar os indicadores de estrutura e de disponibilidade dos imunobiológicos, eles apresentaram variação ao longo dos ciclos, porém, observou-se que ambos os indicadores foram considerados, em média, como bons em todos os ciclos. Constatou-se que a disponibilidade de imunobiológicos aumentou ao longo dos ciclos, sendo boa em 52,8% dos municípios no Ciclo 1 e 68,9% no Ciclo 3, sendo possível observar um aumento significativo desses índices ao longo dos ciclos avaliados (valores-p < 0,05). Já a estrutura boa aumentou do Ciclo 1 para o Ciclo 2, diminuindo no Ciclo 3. E a estrutura regular aumentou no Ciclo 3 (valores-p < 0,05) (Tabela 2).

**Tabela 2:** Indicadores de estrutura das unidades básicas de saúde para imunização, disponibilidade de imunobiológicos e pontuação da cobertura vacinal nos municípios brasileiros segundo os ciclos 1, 2 e 3 do PMAQ. Brasil, 2011 a 2019.

Variável avaliada	Indicador	Ciclo^a^ 1		Ciclo 2		Ciclo 3		Valor-p^b^
		n	%	n	%	n	%	
Estrutura^c^	Ruim	1.343	33,77	826	20,93	705	17,79	< 0,001
	Regular	618	15,54	474	12,01	1.025	25,87	
	Bom	2.016	50,69	2.647	67,06	2.232	56,34	
Disponibilidade de imunobiológicos	Ruim	1.602	40,28	996	25,23	885	22,34	< 0,001
	Regular	272	6,84	685	17,35	347	8,76	
	Bom	2.103	52,88	2.266	57,41	2.730	68,90	
Pontuação cobertura vacinal geral	Muito baixa	585	14,71	347	8,79	770	19,43	< 0,001
	Baixa	2.099	52,78	1.424	36,08	1.525	38,49	
	Adequada	1.293	32,51	2.176	55,13	1.667	42,07	
BCG-ID	Muito baixa	727	18,28	711	18,01	420	10,60	< 0,001
	Baixa	1.280	32,19	983	24,90	1.421	35,87	
	Adequada	1.970	49,53	2.253	57,08	2.121	53,53	
Vacina oral de rotavírus humano	Muito baixa	36	0,91	25	0,63	29	0,73	< 0,001
	Baixa	1.615	40,61	843	21,36	1.161	29,30	
	Adequada	2.326	58,49	3.079	78,01	2.772	69,96	
Meningocócica C	Muito baixa	44	1,11	15	0,38	28	0,71	< 0,001
	Baixa	1.098	27,61	966	24,47	1.541	38,89	
	Adequada	2.835	71,28	2.966	75,15	2.393	60,40	
Hepatite B	Muito baixa	4	0,10	18	0,46	45	1,14	< 0,001
	Baixa	1.401	35,23	990	25,08	1.750	44,17	
	Adequada	2.572	64,67	2.939	74,46	2.167	54,69	
Tetravalente/ Pentavalente	Muito baixa	26	0,65	18	0,46	48	1,21	< 0,001
	Baixa	2.541	63,89	1.100	27,87	2.116	53,41	
	Adequada	1.410	35,45	2.829	71,67	1.798	45,38	
Pneumocócica 10	Muito baixa	100	2,51	23	0,58	20	0,50	< 0,001
	Baixa	1.848	46,47	1.166	29,54	1.268	32,00	
	Adequada	2.029	51,02	2.758	69,88	2.674	67,49	
Poliomielite	Muito baixa	9	0,23	21	0,53	28	0,71	< 0,001
	Baixa	1.350	33,95	1.064	26,96	1.891	47,73	
	Adequada	2.618	65,83	2.862	72,51	2.043	51,56	
Tríplice viral - D1	Muito baixa	9	0,23	6	0,15	24	0,61	< 0,001
	Baixa	1.369	34,42	682	17,28	1.503	37,94	
	Adequada	2.599	65,35	3.259	82,57	2.435	61,46	

PMAQ: Programa Nacional de Melhoria do Acesso e da Qualidade da Atenção Básica; n: número de municípios correspondente; BCG: bacilo de Calmette e Guérin.

^a^ Ciclo 1 corresponde ao período de 2011 a 2012, o Ciclo 2, de 2013 a 2015, e o Ciclo 3, de 2016 a 2019; ^b^ Teste qui-quadrado; ^c^ Estrutura das unidades básicas de saúde para imunização.

Assim como os indicadores de estrutura e de disponibilidade, os indicadores da frequência das coberturas vacinais, considerados adequados, variaram ao longo dos ciclos (valores-p < 0,0001). No Ciclo 1, foram registrados os menores percentuais de municípios com cobertura vacinal adequada, variando de 35,4% (penta) a 71,2% (meningo C) (Tabela 2). No Ciclo 2, foi visto que as frequências dos municípios com as coberturas vacinais adequadas (de forma geral e por imunobiológico) aumentaram, contudo, houve queda desses indicadores no Ciclo 3 (Tabela 2).

Analisando individualmente as variáveis relacionadas à presença das estruturas das UBS, como serviço de vacinação, sala de vacina, geladeira exclusiva para vacina, caixas térmicas para vacinas e cartão vacinação, observam-se variações significativas de acordo com o indicador de pontuação de cobertura vacinal (valores-p < 0,05), exceto o serviço de vacinação no Ciclo 1 e o cartão de vacinação no Ciclo 3 (Tabela 3). O mesmo fato foi observado considerando a disponibilidade individual dos imunobiológicos nas UBS, com exceção para a vacina oral de rotavírus humano (Tabela 3). Ressalta-se que na maioria dos ciclos, as UBS que sempre tinham os imunobiológicos disponíveis apresentaram maiores indicadores adequados de cobertura vacinal (valores-p < 0,05), com exceção para BCG-ID no Ciclo 1 e da Poliomielite no Ciclo 2. Por fim, no Ciclo 3, a totalidade dos parâmetros avaliados representou a maioria dos municípios, para o indicador de pontuação de cobertura adequado.

**Tabela 3:** Avaliação da estrutura das unidades básicas de saúde para imunização e da disponibilidade de imunobiológicos em relação ao indicador de pontuação de cobertura vacinal geral nos municípios brasileiros, segundo os ciclos de avaliação do PMAQ. Brasil, 2011 a 2019.

Variável avaliada		Indicador de pontuação de cobertura vacinal geral														
		Ciclo^a^ 1				Valor-p	Ciclo 2				Valor-p	Ciclo 3				Valor-p^b^
		Baixa		Adequada			Baixa		Adequada			Baixa		Adequada		
		n	%	n	%		n	%	n	%		n	%	n	%	
Estrutura^c^	Bom	1.050	50,02	708	54,76	0,001	952	66,85	1.519	69,81	< 0,001	877	57,51	870	52,19	< 0,001
Disponibilidade de imunobiológicos		1.085	51,69	734	56,77	0,006	783	54,99	1.326	60,94	< 0,001	1.037	68,00	1.152	69,11	< 0,001
Serviço de vacinação	Sim	1.534	73,08	935	72,31	0,854	1.201	84,34	1.754	80,61	0,015	1.257	82,53	1.331	79,84	< 0,001
Sala de vacina		1.326	63,17	871	67,36	< 0,001	1.131	79,42	1.713	78,72	0,007	1.097	72,03	1.155	69,29	0,008
Geladeira exclusiva para vacina		1.290	61,52	855	66,28	< 0,001	1.072	75,28	1.645	75,60	< 0,001	1.064	69,86	1.073	64,37	< 0,001
Caixas térmicas para vacinas		1.373	65,41	910	70,38	< 0,001	1.138	79,92	1.756	80,70	< 0,001	1.313	86,21	1.388	83,26	0,007
Cartão de vacinação		1.579	75,23	1010	78,11	0,002	1.245	87,43	1.870	85,94	0,286	1.381	90,68	1.460	87,58	< 0,001
BCG-ID	Sempre disponíveis	1.265	39,73	683	47,18	< 0,001	677	52,46	917	57,86	< 0,001	860	43,53	759	54,47	< 0,001
Hepatite B		1.257	59,89	824	63,73	0,001	1.077	75,63	1.644	75,55	0,002	1.190	78,14	1.273	76,36	< 0,001
Meningocócica C		1.226	58,41	791	61,18	0,051	1.071	75,21	1.634	75,09	0,001	1.162	76,30	1.236	74,15	< 0,001
Poliomielite		1.244	59,27	815	63,03	0,005	411	28,86	731	33,59	< 0,001	1.121	73,60	1.207	72,41	0,016
Pneumocócica 10		1.197	57,03	778	60,17	0,007	1.044	73,31	1.588	72,98	< 0,001	1.176	77,22	1.264	75,82	< 0,001
Tríplice viral		1.144	54,50	762	58,93	0,002	1.057	74,23	1.622	74,54	0,001	1.175	77,15	1.247	74,81	< 0,001
Tetravalente Pentavalente		1.123	53,50	754	58,31	0,008	1.078	75,70	1.635	75,14	0,005	1.152	75,64	1.232	73,91	0,013
Vacina oral de rotavírus humano		1.241	59,12	810	62,65	0,015	1.073	75,35	1.641	75,41	< 0,001	1.067	70,06	1.176	70,55	0,221

PMAQ: Programa Nacional de Melhoria do Acesso e da Qualidade da Atenção Básica; n: número de municípios correspondente; BCG: bacilo de Calmette e Guérin.

^a^ Ciclo 1 corresponde ao período de 2011 a 2012, o Ciclo 2, de 2013 a 2015, e o Ciclo 3, de 2016 a 2019; ^b^ Teste de Kruskal-Wallis; ^c^ Estrutura das unidades básicas de saúde para imunização.

Houve associação entre pontuação da cobertura vacinal (classificada como muito baixa, baixa e adequada) nos municípios, nas unidades federativas e nas regiões de ocorrência (valores-p < 0,001). O comportamento no nível adequado foi igual entre todas as regiões, ou seja, aumento no Ciclo 1 para o Ciclo 2 e redução do Ciclo 2 para o Ciclo 3 (Tabela 4). As regiões que apresentaram os maiores indicadores de pontuação da cobertura vacinal nos municípios classificados como adequados foram: Sul, Sudeste e Centro-Oeste (Tabela 4).

**Tabela 4:** Avaliação do indicador de pontuação da cobertura vacinal nos municípios (muito baixa, baixa e adequada) segundo as regiões e estados do Brasil ao longo dos ciclos de avaliação do PMAQ. Brasil, 2011 a 2019.

Variável		Indicador de pontuação da cobertura vacinal geral																				
		Ciclo^a^ 1						Valor-p	Ciclo 2						Valor-p	Ciclo 3						Valor-p^b^
		Muito baixa		Baixa		Adequada			Muito baixa		Baixa		Adequada			Muito baixa		Baixa		Adequada		
		n	%	n	%	n	%		n	%	n	%	n	%		n	%	n	%	n	%	
Região	Centro-Oeste	35	9,38	177	47,45	161	43,16	< 0,001	12	3,23	106	28,49	254	68,28	< 0,001	76	20,32	134	35,83	164	43,85	< 0,001
	Nordeste	297	23,80	711	56,97	240	19,23		225	18,01	568	45,48	456	36,51		370	29,58	494	39,49	387	30,94	
	Norte	52	20,63	136	53,97	64	25,40		32	12,85	108	43,37	109	43,78		64	24,90	95	36,96	98	38,13	
	Sudeste	116	9,53	631	51,85	470	38,62		39	3,24	399	33,11	767	63,65		168	13,90	460	38,05	581	48,06	
	Sul	85	9,58	444	50,06	358	40,36		39	4,47	243	27,87	590	67,66		92	10,56	342	39,27	437	50,17	
Estado	AC	2	33,33	4	66,67	0	0,00	< 0,001	3	50,00	3	50,00	0	0,00	< 0,001	4	66,67	2	33,33	0	0,00	< 0,001
	AL	19	23,46	52	64,20	10	12,35		10	12,35	38	46,91	33	40,74		7	8,64	38	46,91	36	44,44	
	AM	12	52,17	11	47,83	0	0,00		3	13,04	15	65,22	5	21,74		12	52,17	8	34,78	3	13,04	
	AP	2	20,00	5	50,00	3	30,00		5	50,00	2	20,00	3	30,00		5	50,00	4	40,00	1	10,00	
	BA	76	25,25	170	56,48	55	18,27		55	18,33	133	44,33	112	37,33		145	48,33	90	30,00	65	21,67	
	CE	6	4,48	66	49,25	62	46,27		0	0,00	14	10,45	120	89,55		4	2,99	39	29,10	91	67,91	
	ES	1	2,13	28	59,57	18	38,30		0	0,00	8	17,02	39	82,98		4	8,51	19	40,43	24	51,06	
	GO	17	8,10	98	46,67	95	45,24		8	3,81	73	34,76	129	61,43		38	18,10	88	41,90	84	40,00	
	MA	9	28,13	21	65,63	2	6,25		4	12,50	13	40,63	15	46,88		10	31,25	13	40,63	9	28,13	
	MG	81	11,71	341	49,28	270	39,02		24	3,50	211	30,80	450	65,69		80	11,58	243	35,17	368	53,26	
	MS	2	3,57	31	55,36	23	41,07		1	1,79	4	7,14	51	91,07		15	26,32	14	24,56	28	49,12	
	MT	16	14,95	48	44,86	43	40,19		3	2,83	29	27,36	74	69,81		23	21,50	32	29,91	52	48,60	
	PA	14	20,00	44	62,86	12	17,14		9	13,24	42	61,76	17	25,00		35	52,24	22	32,84	10	14,93	
	PB	84	42,42	105	53,03	9	4,55		48	24,24	107	54,04	43	21,72		43	21,72	113	57,07	42	21,21	
	PE	24	16,00	90	60,00	36	24,00		12	8,00	81	54,00	57	38,00		19	12,67	78	52,00	53	35,33	
	PI	44	28,21	99	63,46	13	8,33		71	44,10	78	48,45	12	7,45		68	42,24	54	33,54	39	24,22	
	PR	16	5,50	137	47,08	138	47,42		2	0,69	71	24,48	217	74,83		24	8,33	102	35,42	162	56,25	
	RJ	2	3,17	45	71,43	16	25,40		1	1,61	20	32,26	41	66,13		7	11,11	37	58,73	19	30,16	
	RN	33	23,74	83	59,71	23	16,55		22	16,06	86	62,77	29	21,17		63	45,32	47	33,81	29	20,86	
	RO	1	3,45	19	65,52	9	31,03		1	3,23	11	35,48	19	61,29		0	0,00	10	31,25	22	68,75	
	RR	4	66,67	2	33,33	0	0,00		0	0,00	5	83,33	1	16,67		1	16,67	2	33,33	3	50,00	
	RS	57	16,96	169	50,30	110	32,74		24	7,45	84	26,09	214	66,46		47	14,46	126	38,77	152	46,77	
	SC	12	4,62	138	53,08	110	42,31		13	5,00	88	33,85	159	61,15		21	8,14	114	44,19	123	47,67	
	SE	2	3,51	25	43,86	30	52,63		3	5,36	18	32,14	35	62,50		11	19,64	22	39,29	23	41,07	
	SP	32	7,71	217	52,29	166	40,00		14	3,41	160	38,93	237	57,66		77	18,87	161	39,46	170	41,67	
	TO	17	15,74	51	47,22	40	37,04		11	10,48	30	28,57	64	60,95		7	6,19	47	41,59	59	52,21	

PMAQ: Programa Nacional de Melhoria do Acesso e da Qualidade da Atenção Básica; n: número de municípios correspondente; AC (Acre); AL (Alagoas); AM (Amazonas); AP (Amapá); BA (Bahia); CE (Ceará); ES (Espírito Santo); GO (Goiás); MA (Maranhão); MG (Minas Gerais); MS (Mato Grosso do Sul); MT (Mato Grosso); PA (Pará); PB (Paraíba); PE (Pernambuco); PI (Piauí); PR (Paraná); RJ (Rio

de Janeiro); RN (Rio Grande do Norte); RO (Rondônia); RR (Roraima); RS (Rio Grande do Sul); SC (Santa Catarina); SE (Sergipe); SP (São Paulo); TO (Tocantins).

^a^ Ciclo 1 corresponde ao período de 2011 a 2012, o Ciclo 2, de 2013 a 2015, e o Ciclo 3, de 2016 a 2019.

^b^ Testes qui-quadrado e exato de Fisher.

No Ciclo 1, somente Sergipe teve mais de 50,0% dos municípios com esse indicador adequado. No Ciclo 2, 14 estados apresentaram a maioria dos seus municípios com pontuação de cobertura adequada, com destaque para o Mato Grosso do Sul (91,07%). Por fim, no Ciclo 3, somente sete estados contaram com 50,0% ou mais dos municípios com o indicador adequado, sendo a maior frequência vista em Rondônia (68,75%) (Tabela 4). Ao observar a nível municipal, pode-se destacar que ao longo dos ciclos do PMAQ avaliados, nenhum município do Acre apresentou indicador de pontuação da cobertura vacinal adequada, bem como os municípios do Amazonas e Roraima (Ciclo 1).

Ao averiguar a relação dos indicadores de estrutura para imunização e disponibilidade de imunobiológicos e seus efeitos na cobertura vacinal (adequada ou não adequada), observou-se que melhorar a estrutura das UBS ou a disponibilidade de imunobiológicos resulta em maior cobertura vacinal (Tabela 5). Mantendo o indicador “disponibilidade do imunobiológico” fixo (bom), a chance de ter cobertura adequada é de 86,28% (= 3,22% + 83,06%) maior do que a cobertura não adequada para um indicador de estrutura bom em relação ao ruim (valor-p < 0,05). Já quanto à disponibilidade boa em relação à disponibilidade ruim, mantendo o indicador “estrutura” fixo (bom), a chance de ter cobertura adequada é de 48,3% (= -34,76% + 83,06%), maior do que a cobertura não adequada para um indicador “disponibilidade do imunobiológico” bom em relação ao indicador “disponibilidade do imunobiológico” ruim (valor-p < 0,05) (Tabela 5).

**Tabela 5:** Análise da influência das unidades básicas de saúde e imunobiológicos na cobertura vacinal. Brasil, 2011 a 2019.

Equações de estimações generalizadas							
Variável	β	Erro Padrão	Exp(β)	Alteração	IC95% Exp(β)	IC95% Alteração	Valor-p
EST = Ruim	-	-	1,00	-	-	-	-
EST = Regular	-0,25	0,10	0,78	-22,07%	(0,639 a 0,95)	(-36,1% a -4,95%)	0,014
EST = Bom	0,03	0,13	1,03	3,22%	(0,793 a 1,343)	(-20,66% a 34,3%)	0,813
DI = Ruim	-	-	1,00	-	-	-	-
DI = Regular	-0,19	0,36	0,83	-16,94%	(0,412 a 1,675)	(-58,81% a 67,51%)	0,604
DI = Bom	-0,43	0,27	0,65	-34,76%	(0,385 a 1,106)	(-61,51% a 10,58%)	0,113
EST = Regular* DI = Regular	0,44	0,38	1,55	55,05%	(0,731 a 3,291)	(-26,94% a 229,06%)	0,253
EST = Bom* DI = Regular	0,21	0,38	1,24	23,55%	(0,583 a 2,618)	(-41,69% a 161,77%)	0,581
EST = Regular* DI = Bom	0,71	0,29	2,03	102,76%	(1,147 a 3,584)	(14,71% a 258,39%)	0,015
EST = Bom* DI = Bom	0,60	0,30	1,83	83,06%	(1,016 a 3,297)	(1,64% a 229,69%)	0,044
**Estimativas marginais das médias**							
**Indicador de disponibilidade de imunobiológicos**	**Indicador de estrutura das UBS**		**Estimativa**		**Erro**	**Valor-p**	
Ruim - Regular	Estrutura = Ruim		0,186		0,358	0,604	
Ruim - Bom	Estrutura = Ruim		0,427		0,269	0,113	
Regular - Bom	Estrutura = Ruim		0,242		0,448	0,590	
Ruim - Regular	Estrutura = Regular		-0,253		0,143	0,077	
Ruim - Bom	Estrutura = Regular		-0,280		0,108	0,009	
Regular - Bom	Estrutura = Regular		-0,027		0,122	0,826	
Ruim - Regular	Estrutura = Bom		-0,026		0,144	0,857	
Ruim - Bom	Estrutura = Bom		-0,178		0,131	0,175	
Regular - Bom	Estrutura = Bom		-0,152		0,068	0,026	

UBS: unidade básica de saúde; IC95%: intervalo de confiança de 95%; EST: estrutura das unidades básicas de saúde para imunização; DI: disponibilidade de imunobiológico.

Por meio dos contrastes, considerando a estrutura regular, nota-se que a disponibilidade boa de imunobiológicos favorece significativamente a cobertura vacinal em comparação com a disponibilidade ruim (valor-p < 0,01). Para as UBS com estrutura boa, a disponibilidade tanto no nível regular quanto bom de imunobiológicos aumenta a cobertura vacinal (Tabela 5).

## DISCUSSÃO

A Política Nacional de Atenção Básica (PNAB) enfatiza a importância da conformidade da infraestrutura nas UBS (sendo recomendada a existência de sala de vacina), as quais devem seguir as normas sanitárias, além de contar com a ambiência apropriada, equipamentos adequados, recursos humanos capacitados e materiais e insumos suficientes para a atenção à saúde^29^. Isso é fundamental para garantir condições adequadas para o pleno funcionamento dessas unidades. No estudo, a análise de indicadores de estrutura demostrou que, de forma geral, houve melhorias nas estruturas das UBS municipais entre os ciclos do PMAQ avaliados (Ciclo 1 – de 2011 a 2012, Ciclo 2 – de 2013 a 2015 e Ciclo 3 – de 2016 a 2019). Variações nas estruturas das UBS já haviam sido observadas^15,17^, inclusive, considerando ciclos de avaliações^7,17^, e podem ser explicadas por diferentes fatores, como: investimentos em infraestrutura^17^, mudanças nas diretrizes^17,18^e políticas de saúde^22,29^. Os mesmos fatores estão relacionados com as melhorias observadas na disponibilidade dos imunobiológicos nas UBS municipais ao longo dos ciclos do PMAQ avaliados^8^.

A disponibilidade dos imunobiológicos está ligada à oportunidade de vacinação e é influenciada por vários fatores como: condições ideias de conservação, produção e logística de distribuição^8,9,16^. Estima-se que um terço das crianças não receberam as vacinas do calendário básico do primeiro ano de vida em países de baixa e média renda, o que foi uma oportunidade perdida de vacinação. Assim, é importante observar separadamente a disponibilidade de cada imunobiológico avaliado, neste estudo, bem como as suas condições de conservação (geladeiras e/ou de caixas térmicas). Ao averiguar este item para cada imunobiológico, notou-se uma elevada disponibilidade nos Ciclos 2 e 3, com valores superiores a 70,0%, exceto para a vacina BCG-ID que apresentou baixa disponibilidade em todos os ciclos avaliados (< 55,0%), e resultados similares foram observados em outros estudos^8,19^.

Apesar do aumento significativo da disponibilidade das vacinas ao longo dos ciclos neste estudo, é importante ressaltar que foi observada queda na disponibilidade da maioria das vacinas do calendário vacinal infantil^8^, fato que compromete tanto a saúde da criança quanto da população em geral^8,30^. Cabe destacar que “a irregularidade no fornecimento dos imunobiológicos decorrentes de problemas de produção, tanto relacionados ao processo produtivo dos laboratórios públicos quanto dos privados, identificada nos últimos anos”^1^ pode influenciar o monitoramento do ICV, seja pela falta de vacinação oportuna ou, ainda, mesmo que o desabastecimento seja regularizado, a depender a faixa etária, a dose administrada em ocasião posterior (fora da faixa etária específica) não contará para os cálculos dos indicadores^1^. A inclusão de novas vacinas ao longo do tempo no calendário vacinal do país também pode influenciar a disponibilidade desses imunobiológicos e sua cobertura, considerando o tempo até que os serviços de vacinação em todo o território tenham acesso a esses produtos, uma vez que a partir do ano 2006, “se observa uma crescente incorporação de novas vacinas no PNI”^1^.

Em relação aos ICV, foram observados municípios com maiores ICV no Ciclo 2, seguidos por significativa queda, resultando em patamares abaixo das metas de vacinação preconizadas pelo Ministério da Saúde que podem ser vistos a partir de 2016^1^. A redução do ICV é motivo de preocupação por estar associada ao aumento da incidência de doenças imunopreveníveis. Um exemplo disso é o retorno do sarampo, doença da qual o Brasil havia sido declarado livre desde 2016, mas que em 2018 registrou mais de 10 mil casos^1^. Acredita-se que essa queda na vacinação durante o Ciclo 3 esteja relacionada principalmente a fatores socioeconômicos adversos^14^, à disseminação de notícias falsas^15^, ao movimento antivacina^12,16^e à diminuição da ênfase na prevenção, devido à redução na incidência de várias doenças^1^, assim como da estrutura das UBS e da sua disponibilidade de imunobiológicos^1,8,9^.

A adequação da estrutura da UBS apresentou associação com maiores ICV, fato este que corrobora com resultados de outros estudos^7,14,15,30^, bem como a disponibilidade dos imunobiológicos^8,9,15^. Curiosamente, quando as estruturas das UBS municipais são consideradas regulares, observa-se redução na cobertura vacinal. Esses resultados divergem das expectativas e indicam a necessidade de investigações mais aprofundadas sobre os determinantes da cobertura vacinal e sua relação com a estrutura e disponibilidade de imunobiológicos nas UBS, uma vez que por mais que a situação é regular, deveria ser melhor que a estrutura ruim. No entanto, ao avaliar a associação desses fatores de forma conjunta quanto aos ICV, observa-se que, quando a disponibilidade dos imunobiológicos é classificada como boa e a estrutura da UBS como regular ou boa, há aumento significativo da cobertura vacinal. Isso sugere que a combinação desses fatores pode ter impacto positivo na promoção da vacinação e na melhoria da saúde da população, destacando a importância de analisá-los em conjunto para compreender melhor os determinantes da cobertura vacinal.

Ao considerar o ICV segundo as regiões do Brasil, percebe-se que os incrementos nos ICV ao longo dos ciclos avaliados nas regiões Norte, Centro-Oeste e Nordeste já haviam sido observados na literatura^8^. Esses aumentos foram associados à maior disponibilidade de vacina e iniciativas governamentais para a redução na iniquidade entre os serviços de saúde^8^. Historicamente essas regiões são as de menores coberturas vacinais e as causas estão frequentemente associadas aos indicadores sociais e de desenvolvimento^31^. Por outro lado, acredita-se que a redução nos ICV, no mesmo período, nas regiões Sul e Sudeste deve-se principalmente à disseminação de notícias falsas^12^e ao movimento antivacina^12,16^. Esses fatores contribuem para a diminuição da confiança na vacinação e podem impactar negativamente os esforços para manter altas taxas de cobertura vacinal nessas regiões.

É importante ressaltar alguns limites deste estudo. Em relação às coberturas vacinais disponibilizadas pelo SI-PNI, podem existir problemas de registros, com coberturas sub ou superestimadas. As coberturas municipais do SI-PNI podem incluir serviços fora das UBS. A análise do PMAQ se restringiu às estruturas de imunização localizadas dentro das UBS que aderiram ao programa e que apresentaram participação acima de 80,0% das unidades/equipes em pelo menos dois ciclos do PMAQ, as quais, na maioria das vezes, são consideradas de melhor qualidade^19,32^. Além disso, as informações referentes às estruturas de imunização centralizadas nos municípios não foram consideradas. Portanto, é necessário ter cautela ao generalizar os resultados obtidos para todos os serviços de APS no Brasil, visto que, somente em 2019, havia mais de 43 mil equipes de saúde da família (ESF) distribuídas em 5.476 municípios brasileiros (cerca de 0,98 ESF/município)^33^.

Diante do exposto, conclui-se que houve melhorias na estrutura das UBS municipais avaliadas e aumento na disponibilidade de imunobiológicos ao longo do tempo. Os indicadores de cobertura vacinal nos municípios variaram ao longo dos anos e de acordo com as regiões/estados do Brasil. Percebeu-se que maiores indicadores foram observados quando houve combinação de boa disponibilidade de imunobiológicos e estrutura regular ou boa nas UBS, destacando a importância de investigações mais abrangentes sobre os determinantes da vacinação nos diferentes territórios, a melhoria da qualidade e o papel da atenção primária para promover não só a imunização, como também a redução de desigualdades em saúde.
